# Support vector machine-based classification of bulimia nervosa using diffusion tensor imaging

**DOI:** 10.3389/fpsyt.2025.1667996

**Published:** 2025-09-10

**Authors:** Linli Zheng, Yu Wang, Ma Jing, Meiou Wang, Yang Liu, Jin Li, Tao Li, Lan Zhang

**Affiliations:** ^1^ Mental Health Center, West China Hospital, Sichuan University, Chengdu, China; ^2^ Chengdu Fourth People's Hospital, Chengdu, Sichuan, China; ^3^ Department of Neurobiology, Zhejiang University School of Medicine Affiliated Mental Health Centre & Hangzhou Seventh People's Hospital, Hangzhou, Zhejiang, China

**Keywords:** bulimia nervosa, diffusion tensor magnetic resonance image, machine learning, support vector machines, eating disorders

## Abstract

**Background:**

Alterations in brain structure have been suggested to be associated with bulimia nervosa (BN). This study aimed to employ machine learning (ML) methods based on diffusion tensor imaging (DTI) to facilitate the diagnosis of BN and to identify potential neurobiological markers.

**Methods:**

Thirty-four drug-naive females with bulimia nervosa (BN) and 34 age- and gender-matched healthy controls (HCs) underwent diffusion tensor imaging (DTI) scanning. The extracted features included fractional anisotropy (FA), axial diffusivity (AD), radial diffusivity (RD), and mean diffusivity (MD). Support vector machines (SVM), a commonly used machine learning (ML) approach, were employed to distinguish individuals with BN from healthy controls.

**Results:**

Five ML models were constructed. The FA model (AUC=0.821) and the combined FA+MD+AD+RD model (AUC=0.739) exhibited satisfactory classification performance, with the FA model exhibiting the most effective results. The FA model achieved an accuracy of 82.35%, a specificity of 82.35%, and a sensitivity of 85.29%. The contributing brain regions were primarily located in the frontal lobe, brainstem, temporal lobe, midbrain, cerebellar tonsil, and posterior cerebellar lobe. In contrast, the MD model (AUC=0.689), the AD model (AUC=0.621), and the RD model (AUC=0.625) demonstrated poor classification performance.

**Conclusions:**

This study demonstrated that DTI-based machine learning (ML) approaches could effectively differentiate individuals with bulimia nervosa (BN) from healthy controls (HCs), thereby providing insights into potential neurobiological markers associated with BN.

## 1 Introduction

Bulimia nervosa (BN) is characterized by recurrent episodes of impulsive and uncontrollable binge eating, followed by compensatory behaviors such as self-induced vomiting, use of laxatives, or excessive exercise to prevent weight gain. BN typically begins during adolescence or early adulthood and occurs more frequently in females. The overall incidence among women has been reported to be as high as 3%, with an estimated mortality rate ranging from 2.33% to 3.00% (1–3).

Current diagnostic approaches for BN are still largely based on subjective self-reports and clinical evaluations. The notable absence of validated objective biomarkers presents significant challenges in diagnostic accuracy. Therefore, a critical need remains to identify and validate reliable biomarkers to improve diagnosis and guide treatment.

Diffusion tensor imaging (DTI) assesses the structural integrity of the white matter of the brain and indirectly realizes three-dimensional reconstruction of nerve fiber bundles (4, 5). DTI has four parameters: fractional anisotropy (FA), axial diffusivity (AD), radial diffusivity (RD), and mean diffusivity (MD). These parameters reflect the structural integrity of the white matter of the brain and indicate the level of myelination and axonal damage in specific areas (6, 7). High FA values typically indicate well-organized, intact white matter tracts, suggesting efficient communication between brain regions. AD measures water diffusion along the main axis of the nerve fibers. It is thought to be related to axonal integrity; reductions in AD may suggest axonal damage. RD reflects water diffusion perpendicular to the axon. Increased RD is often associated with demyelination, indicating that the insulating myelin sheath around nerve fibers may be damaged. MD represents the average rate of water diffusion in all directions within a tissue. Higher MD values may indicate less dense or more damaged tissue, suggesting abnormalities in brain microstructure. DTI has been gradually applied in the mental disorder field, including schizophrenia, autism, and eating disorders (8, 9).

DTI plays an important role in identifying the biological signs of diseases and abnormal microstructures (10). Previous DTI studies have found differences in the white matter of the brain regions of patients with BN and normal people. Mettler et al. found that DTI images of the bilateral radiating crowns, corpus callosum, insular, and fornix had decreased FA values in these patients compared to normal individuals (11). He et al. found that compared to normal individuals, BN patients had reduced white matter FA values in the upper longitudinal tract, subfrontal occipital tract, uncinate tract, prethalamic radiation, and cingulate (12). DTI parameters may serve as a biological marker for distinguishing patients with BN from healthy subjects.

While most previous studies have employed conventional statistical methods, such approaches often limit the full utilization of complex neuroimaging data. In contrast, machine learning (ML) offers advanced analytic capabilities for training predictive models and extracting latent features. ML has increasingly been applied in neuropsychiatric research to facilitate disease classification and identify novel biomarkers (13–15). ML techniques applied to neuroimaging data include image segmentation, feature extraction, data integration, model development, and validation (13). Support vector machine (SVM), a supervised ML algorithm, is particularly effective for disease classification in small sample studies (16, 17) and has been successfully applied to diagnostic tasks.

To date, few studies have applied ML methods for BN diagnosis. Cerasa et al. used SVM-based ML techniques to analyze structural brain features, achieving an accuracy of 80%, specificity of 72%, and sensitivity of 96% (18). However, the sample size was limited (17 BN patients and 17 controls). Marilyn et al. applied a multi-class ML approach to classify BN, subclinical BN, and healthy individuals using functional MRI data, achieving a maximum accuracy of 62.7%. Their findings implicated reduced frontostriatal activity as a potential neural marker of binge eating (19). Despite these promising results, prior studies were limited by small sample sizes and did not incorporate DTI-derived features.

To improve the diagnostic accuracy of BN and explore reliable neuroimaging biomarkers, four DTI-derived parameters were selected as input features, and SVM was employed to construct classification models distinguishing individuals with BN from healthy controls.

## 2 Materials and methods

### 2.1 Participants

This study included 34 drug-naïve women with BN and age-matched female healthy controls (HCs). All participants, aged 14–30 years and right-handed, were assessed by two psychiatrists using a structured clinical interview for DSM-V disorders (SCID). Patients with BN were recruited from the Mental Health Center of West China Hospital, Sichuan University, and had a history of engaging in binge eating and compensatory behaviors at least once a week during the three months prior to enrollment in the study. Patients were excluded if they had severe physical diseases such as neurogenic diseases, endocrine diseases, or metabolic disorders. Additionally, comorbidity with other major psychiatric disorders, such as schizophrenia, bipolar disorder, major depressive disorder, alcohol or drug abuse, or history of loss of consciousness, was also an exclusion criterion.

HCs were recruited from the community through public advertisements. A total of 43 individuals were initially recruited, but one participant was excluded due to a family history of mental illness, and eight individuals refused to participate due to concern potential side effects of the magnetic resonance imaging (MRI) scan.

The study was approved by the Ethics Committee of West China Hospital of Sichuan University. The study were carried out in accordance with relevant guidelines and regulations. This study was carried out in compliance with Declaration of Helsinki and Good Clinical Practice guidelines. All participants provided written informed consent. Participants under 18 years had obtained written informed consent from a parent and/or legal guardian.

### 2.2 Data acquisition

DTI magnetic resonance (MR) images were obtained using a Philips 3.0T equipment. The method of MRI data acquisition and scanning parameters have been described previously (20, 21). During MRI, all subjects were asked to relax, to keep their eyes closed without falling asleep, to keep their head as motionless as possible, and not to think of anything. All scans were reviewed by a practicing neuroradiologist to exclude gross brain abnormalities.

### 2.3 Image processing and feature extraction

All images were processed using FSL5.0.9 software, including head movement and eddy current correction and gradient direction correction. Subsequently, we selected the range of tensors that needed to be calculated according to the b0 image to obtain the FA, MD, AD, and RD images of each subject. By nonlinear registration, all of the FA, MD, AD, and RD images were transformed into Montreal Neurological Institute (MNI) space, and FMRIB58_FA was used as a template. Thereafter, the mean values of FA, MD, AD, and RD were extracted using JHU-ICBM-tracts-maxprob-thr25 as the brain map.

### 2.4 Machine learning classification

We employed a validated supervised ML method for the differential diagnosis of BN. The SVM algorithm was used to perform classification. The ML process was implemented in LIBSVM, MATLAB2013b, and FSL5.0.9 software. The ML process was achieved using LIBSVM, MATLAB2013b, and FSL5.0.9 software. For the mL analysis, we chose FA, MD, AD and RD and a combination of four parameters.

#### 2.4.1 Feature selection

Individual subject images were first registered to their respective templates and smoothed with a 3mm full-width-at-half-maximum (FWHM) Gaussian kernel. Group differences were assessed using two-sample t-tests on the smoothed images, with a voxel-wise threshold of P < 0.001 (family-wise error [FWE]-corrected). For each statistically significant voxel, a spherical region of interest (ROI) with a 3mm radius was defined, and the mean value of all voxels within this ROI was extracted as the representative feature value.

#### 2.4.2 ML classification

Data were normalized, and the characteristic value was mapped to 0–1. According to the correlation coefficient between each feature and the label, the features within the top 5% of the absolute value of the correlation coefficient were retained as the training object.

The linear kernel SVM method was used for the training. We used leave-one-out cross-validation, repeating the process 68 times by holding out one subject as the test set and using the remaining 67 for training in each iteration. The test set was predicted according to the trained model; the accuracy, specificity, and sensitivity of each training were averaged to obtain the final value; and the corresponding receiver operating characteristic curve, area under the curve (AUC), and contributing brain area were obtained.

### 2.5 Statistical analysis

SPSS statistics version 20.0 (IBM Corp., Armonk, N.Y., USA) was used to perform statistical analysis on subjects’ age, BMI, course of disease, and years of education. The two groups of measurement data were compared using Student’s t-test, and the difference was considered statistically significant at p < 0.05.

## 3 Results

### 3.1 Clinical demographics

The study included 68 female subjects, including 34 BN patients and 34 HCs. There were no statistically significant differences in age, years of education, or BMI between BN and HCs (**Table 1**).

**Table 1:** Comparison of demographic data between bulimia nervosa and healthy controls.

	BN (N=34) Mean ± SD	HCs (N=34) Mean ± SD	T	*P* value
Age (year)	23.50 ± 5.41	22.35 ± 2.52	1.121	0.268
BMI (kg/m^2^)	20.74 ± 2.78	20.21 ± 1.80	0.942	0.350
education (year)	15.85 ± 2.49	15.73 ± 1.44	0.239	0.812
Course of disease (year)	2.91 ± 3.89	—	—	–

Data are presented as mean ± SD. BN, bulimia nervosa; HCs, healthy controls.

### 3.2 ML classification between BN and HCs

#### 3.2.1 ML classification based on FA

**Figure 1:**
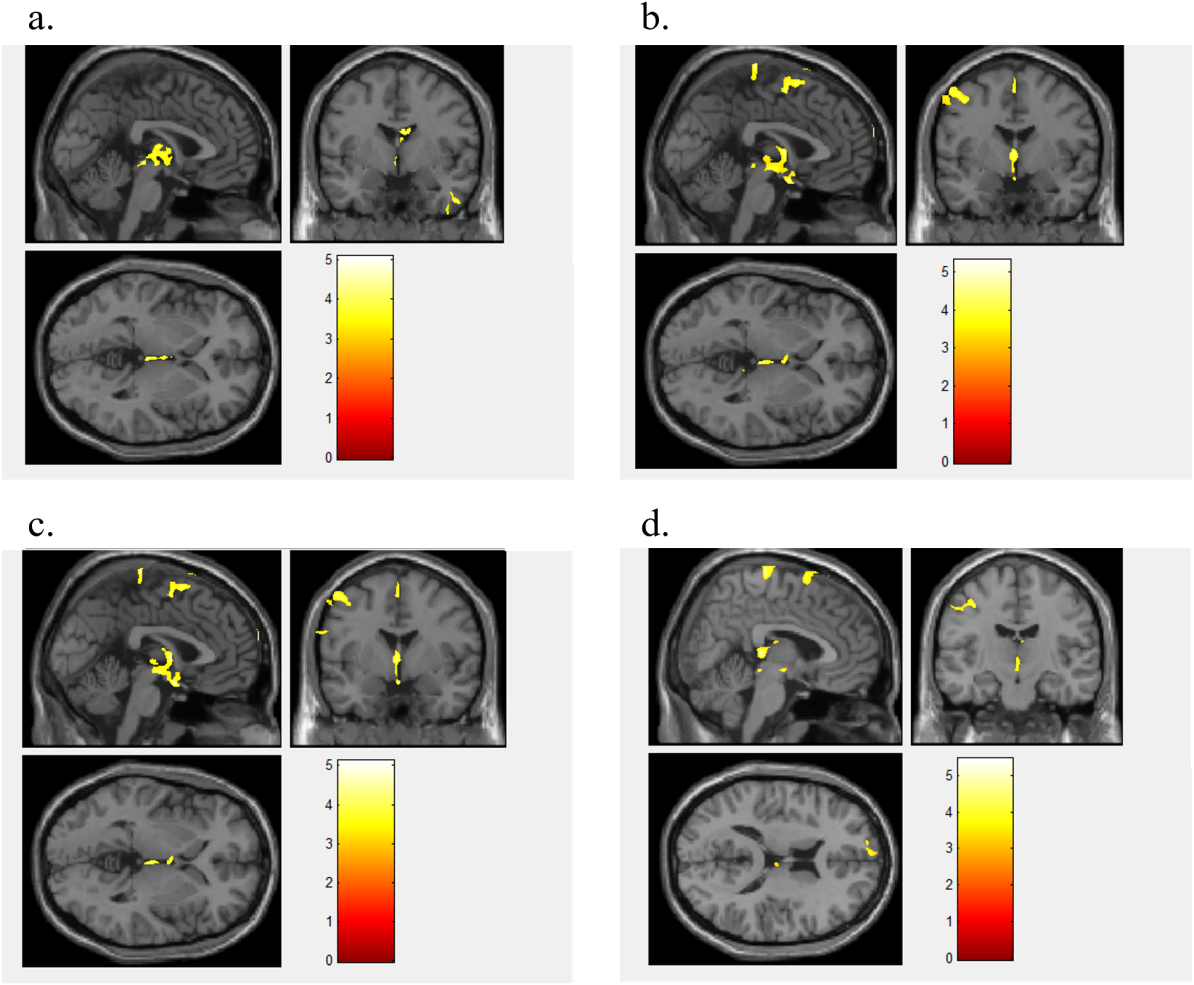
Different brain regions between BN and HC. **Figure 1** shows the different brain regions between BN and HCs for FA value **(a)**, MD value **(b)**, AD value **(c)**, and RD value **(d)**.

**Figure 2:**
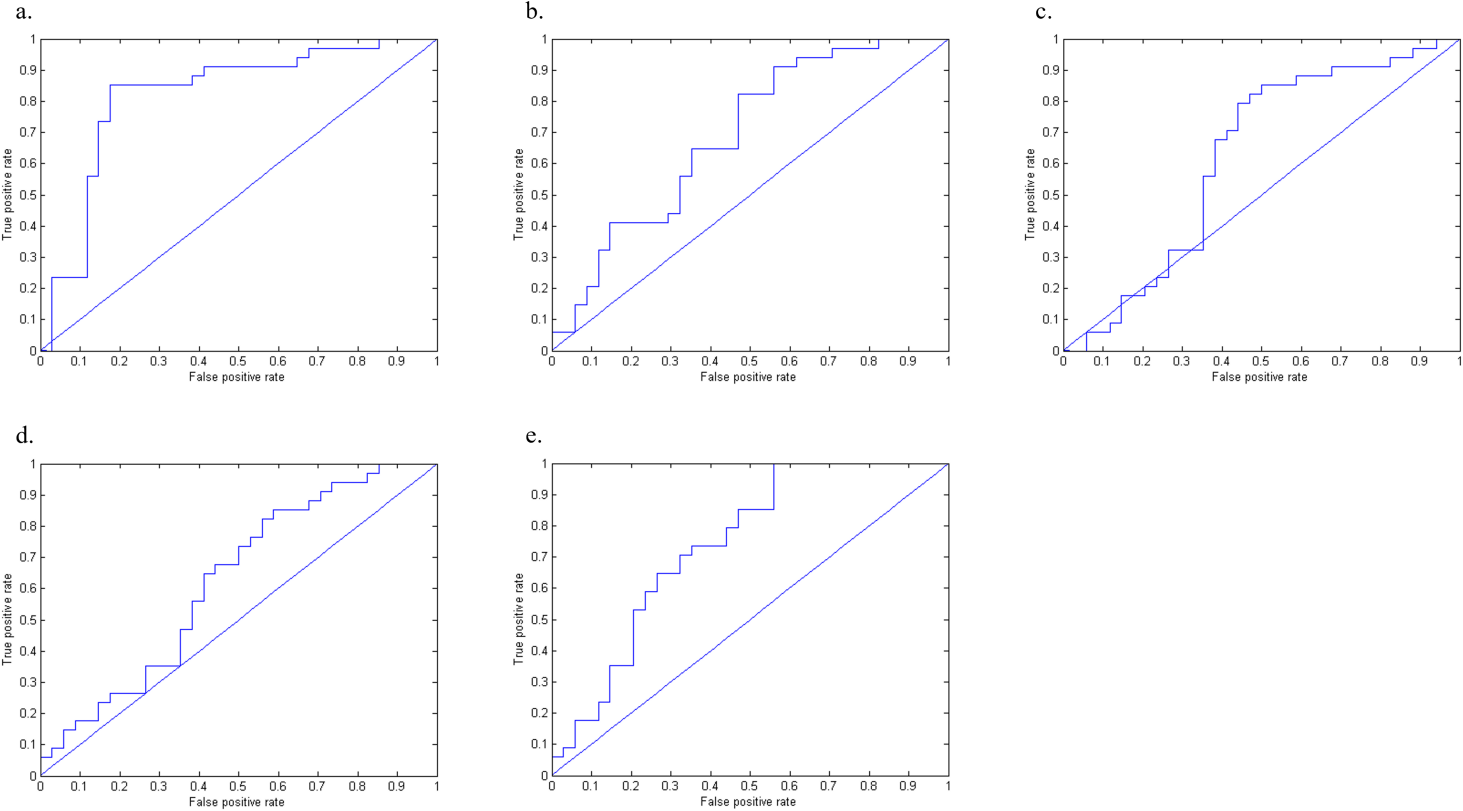
The ROC curve of the machine learning model between BN and HC. **Figure 2** displays the ROC curves of the FA **(a)**, MD **(b)**, AD **(c)**, RD **(d)**, and combined FA+MD+AD+RD **(e)** machine learning models.

When using FA values as features, the brain regions that differed between BN and HCs included the right brainstem, right temporal lobe, right frontal lobe, left inferior occipital gyrus, left midbrain, right middle frontal gyrus, right caudate nucleus, and left middle frontal gyrus (**Figure 1a**). The model showed a high accuracy of 82.35%, specificity of 82.35%, sensitivity of 85.29%, and AUC of 0.821, indicating that the model with the FA value as the data feature had good classification performance (**Figure 2a**).

#### 3.2.2 ML classification based on MD

When using MD value as a data feature, the model demonstrated distinct brain areas of the BN and HCs, which included the left posterior cerebellar lobe, left frontal lobe, left central anterior gyrus, right middle frontal gyrus, left parietal lobe, and right superior frontal gyrus (**Figure 1b**). The accuracy of this model was 63.23%, with a specificity of 52.35%, a sensitivity of 82.35%, and an AUC of 0.689, suggesting that the model utilizing MD as the data feature had relatively weaker classification performance (**Figure 2b**).

#### 3.2.3 ML classification based on AD

When utilizing AD value as a data feature, the model identified distinct brain areas between BN and HCs such as the left cerebellar tonsil, right brain stem, right inferior frontal gyrus, right midbrain, left frontal lobe, left central anterior gyrus, left lateral central posterior gyrus, right middle frontal gyrus, and right medial frontal gyrus (**Figure 1c**). The model exhibited an accuracy of 63.23%, specificity of 55.88%, sensitivity of 79.41%, and an AUC of 0.621, indicating that the model with AD as the data feature had poor classification performance (**Figure 2c**).

#### 3.2.4 ML classification based on RD

When using RD value as a data feature, the model revealed varied brain areas between the BN and HCs including the left posterior cerebellum, right midbrain, left Brodmann area 10, left central anterior gyrus, right frontal lobe, left lateral central posterior gyrus, right superior frontal gyrus, and right medial frontal gyrus (**Figure 1d**). The accuracy of this model was 60.29%, with a specificity of 58.82%, a sensitivity of 64.71%, and an AUC of 0.625, revealing that the classification performance of the model utilizing the RD value as the data feature was poor (**Figure 2d**).

#### 3.3.5 ML classification based on FA+MD+AD+RD ML

Furthermore, the FA, MD, AD, and RD values of the extracted brain regions were combined as features, the same training method was adopted for training. The contributing brain area of this model is a collection of the aforementioned four model brain areas. The model achieved an accuracy of 69.11%, with a specificity of 67.65%, a sensitivity of 70.59%, and an AUC of 0.739 (**Figure 2e**). The classification performance of the model utilizing FA+MD+AD+RD values as data features is acceptable.

## 4 Discussion

In this study, five machine learning (ML) models were constructed to classify bulimia nervosa (BN) patients and healthy controls. Among these, the models based on fractional anisotropy (FA) alone and the combined FA+MD+AD+RD parameters demonstrated acceptable classification performance, with the FA-based model achieving the highest accuracy. Marilyn et al. employed functional magnetic resonance imaging (fMRI) data and a multi-class ML algorithm to differentiate BN patients from healthy controls. Their model achieved maximum specificity and sensitivity values of 67.5% and 62.7%, respectively (19). Compared to prior research, the classification performance achieved in this study surpassed that of earlier ML investigations on BN that utilized alternative brain imaging features.

Our team’s previous two studies, based on diffusion tensor imaging (DTI) combined with machine learning, further elucidated the brain structural differences and classification performance between eating disorder subtypes and healthy controls (22, 23). In distinguishing anorexia nervosa (AN) from healthy controls, the radial diffusivity (RD) model performed best, achieving an AUC of 0.920. Significant brain regions included the hippocampus, brainstem, temporal lobe, and corpus callosum, indicating widespread and pronounced white matter microstructural abnormalities in AN patients. In contrast, for classifying bulimia nervosa (BN) versus healthy controls, the fractional anisotropy (FA) model yielded the best results, with an AUC of 0.821, involving the frontal lobe, temporal lobe, and cerebellum, suggesting relatively milder and more localized white matter abnormalities in BN patients. In our prior research on differentiating AN from BN, the axial diffusivity (AD) model was primarily used, achieving an AUC of 0.793. The key differentiating brain regions were mainly localized in the left middle temporal gyrus and superior temporal gyrus. For BN versus healthy controls, the classification mainly relied on the FA model, with an accuracy of 82.35% and an AUC of 0.821, involving multiple regions including the frontal lobe, temporal lobe, and cerebellum. Comparatively, the classification performance for BN versus healthy controls was superior to that for AN versus BN, suggesting that BN patients show broader and more pronounced brain structural differences compared to healthy individuals. In the AN versus BN classification, the AD model achieved an accuracy of 75.86% and an AUC of 0.793, with differentiating brain regions primarily confined to the left middle and superior temporal gyri, reflecting the neurobiological similarity of these two eating disorder subtypes. The accuracy was notably lower than that for classifying either AN or BN versus healthy controls, consistent with the clinical observation that these two disorders exhibit overlapping symptoms and subtle differences. In summary, AN patients exhibit more widespread and significant brain structural alterations, facilitating clearer distinction from healthy controls; BN patients show relatively limited white matter abnormalities. Differences between AN and BN are minimal, aligning closely with clinical presentations. Future studies should focus on subtle local brain structural changes and integrate multimodal imaging data to further enhance the diagnostic accuracy and clinical intervention specificity for eating disorder subtypes.

The contributing brain regions identified in this study included the frontal lobe, brainstem, temporal lobe, midbrain, cerebellar tonsil, and posterior cerebellar lobe, with the frontal lobe emerging as a common region across different models. The frontal lobe is critically involved in executive functions such as evaluating the consequences of actions, selecting and regulating behaviors, inhibiting socially inappropriate responses, and discerning similarities and differences (24). Clinically, bulimia nervosa (BN) is characterized by recurrent, uncontrolled, and impulsive binge eating. White matter abnormalities within the frontal lobe observed in this study may be associated with impaired self-regulation and conflict resolution behaviors in BN. Previous research has demonstrated that abnormal white matter changes in BN patients predominantly affect fiber tracts traversing the frontal lobe. He et al. reported significant reductions in anisotropy across multiple white matter fiber tracts in BN patients (12), with white matter microstructural abnormalities particularly pronounced within the frontal lobe fiber bundles of severely affected individuals. Functional MRI studies by Marsh et al. revealed that, compared to healthy controls, female BN patients exhibited reduced activation in the frontal lobe, especially in the left inferior and superior frontal gyri, during self-regulation tasks (25). These findings align with previous research.

The orbitofrontal cortex (Brodmann areas 10, 11, and 47) is part of the prefrontal cortex in the frontal lobe. The orbitofrontal cortex receives projections from the dorsal medial nucleus of the thalamus and is involved in emotions during the decision-making process (26). This study found that the FA value of the medial orbitofrontal cortex of patients with BN was decreased, indicating that the white matter integrity of the patients was lower than that of the normal group. Previous studies have also found that the functional connection between the medial orbitofrontal cortex and the left anterior insula is increased in patients with BN (27). Damage to the white matter in this area may lead to excessive activation of the reward center during feeding, leading to overeating. This findings of this study are consistent with the previous studies, and the frontal lobe white matter can be considered as a brain imaging marker for BN.

In current clinical practice, the diagnosis of bulimia nervosa primarily relies on psychiatric evaluation. Patients often exhibit complex behavioral patterns, such as binge eating followed by compensatory behaviors, which typically occur in private settings and are difficult for others to detect. Due to the stigma associated with the illness, patients may conceal their condition, and the clinical assessment based on self-reporting can be biased, resulting in a lack of objectivity in diagnosis. Machine learning models based on neuroimaging can assist in the diagnosis of bulimia nervosa by providing more objective diagnostic evidence. Our research model can be applied in clinical practice to assist in the diagnosis of eating disorders and subtype classification. In future clinical applications, machine learning models may be used to analyze patients’ DTI metrics before and after treatment to evaluate therapeutic efficacy and disease progression. This study identified abnormal brain regions in bulimia nervosa, which may help reveal the biological basis of the disorder and promote further basic research and the development of new therapeutic approaches.

Several limitations were noted in this study. First, the sample size was relatively small, and no independent datasets were included for validation, thereby limiting the ability to assess the robustness and generalizability of the model. Second, only individual DTI imaging data were utilized as features, resulting in limited data diversity. Third, the analysis did not evaluate the correlation between clinical characteristics and brain regions exhibiting significant alterations, hindering a comprehensive understanding of the relationship between specific brain changes and the pathology of bulimia nervosa (BN). Future studies should consider incorporating multimodal imaging data and larger, independent cohorts to enhance model reliability and improve insights into disease mechanisms.

## 5 Conclusion

In this study, the support vector machine (SVM) model based on fractional anisotropy (FA) features derived from diffusion tensor imaging (DTI) data was able to effectively differentiate individuals with bulimia nervosa (BN) from healthy controls (HCs). This finding suggests that the integration of DTI into the diagnostic process may enhance the accuracy and precision of BN diagnosis. Moreover, reduced FA values in the frontal lobe may represent a potential neuroimaging marker for BN. Such markers could contribute to early detection, improve understanding of disease severity, and support the development of more targeted and effective treatment strategies for individuals affected by BN. In future research, functional MRI data and psychological assessment measures should be incorporated as features in multimodal machine learning studies to improve the diagnosis and classification of eating disorders.

## Data Availability

The raw data supporting the conclusions of this article will be made available by the authors, without undue reservation.
